# Phytoplankton-Associated Bacterial Community Composition and Succession during Toxic Diatom Bloom and Non-Bloom Events

**DOI:** 10.3389/fmicb.2016.01433

**Published:** 2016-09-12

**Authors:** Marilou P. Sison-Mangus, Sunny Jiang, Raphael M. Kudela, Sanjin Mehic

**Affiliations:** ^1^Department of Ocean Sciences and Institute of Marine Sciences, University of California, Santa Cruz, Santa Cruz, CAUSA; ^2^Department of Civil and Environmental Engineering, University of California, Irvine, Irvine, CAUSA

**Keywords:** algal bloom, *Pseudo-nitzschia*, diatom, bacteria, *Vibrio*, *Planococcus*, nutrients, domoic acid

## Abstract

*Pseudo-nitzschia* blooms often occur in coastal and open ocean environments, sometimes leading to the production of the neurotoxin domoic acid that can cause severe negative impacts to higher trophic levels. Increasing evidence suggests a close relationship between phytoplankton bloom and bacterial assemblages, however, the microbial composition and succession during a bloom process is unknown. Here, we investigate the bacterial assemblages before, during and after toxic and non-toxic *Pseudo-nitzschia* blooms to determine the patterns of bacterial succession in a natural bloom setting. Opportunistic sampling of bacterial community profiles were determined weekly at Santa Cruz Municipal Wharf by 454 pyrosequencing and analyzed together with domoic acid levels, phytoplankton community and biomass, nutrients and temperature. We asked if the bacterial communities are similar between bloom and non-bloom events and if domoic acid or the presence of toxic algal species acts as a driving force that can significantly structure phytoplankton-associated bacterial communities. We found that bacterial diversity generally increases when *Pseudo-nitzschia* numbers decline. Furthermore, bacterial diversity is higher when the low-DA producing *P. fraudulenta* dominates the algal bloom while bacterial diversity is lower when high-DA producing *P. australis* dominates the algal bloom, suggesting that the presence of algal toxin can structure bacterial community. We also found bloom-related succession patterns among associated bacterial groups; Gamma-proteobacteria, were dominant during low toxic *P. fraudulenta* blooms comprising mostly of *Vibrio* spp., which increased in relative abundance (6–65%) as the bloom progresses. On the other hand, Firmicutes bacteria comprising mostly of *Planococcus* spp. (12–86%) dominate during high toxic *P. australis* blooms, with the bacterial assemblage showing the same bloom-related successional patterns in three independent bloom events. Other environmental variables such as nitrate and phosphate and temperature appear to influence some low abundant bacterial groups as well. Our results suggest that phytoplankton-associated bacterial communities are strongly affected not just by phytoplankton bloom in general, but also by the type of algal species that dominates in the natural bloom.

## Introduction

Phytoplankton plays an important role in global carbon cycling by consuming carbon dioxide from the atmosphere for photosynthesis and sequestering the fixed carbon as cells sink down into the deep ocean. Phytoplankton bloom, happening either in the coast, upwelling regions or open ocean, not only provides organic materials to the higher trophic food web but also provides photosynthate-released dissolved organic matter (Larsson and Hagstrom, 1979) and organic suspended matter for bacterial attachment and subsequent degradation by heterotrophic bacterial communities (Smith et al., 1995). Azam et al. (1983) reported that bacterial biomass and production is tightly coupled with phytoplankton biomass. With the use of molecular tools, many workers discovered that bacterial communities change in composition as algal bloom peak and decline (Riemann et al., 2000; Teeling et al., 2012; Klindworth et al., 2014). Bacterial groups such as Alpha-proteobacteria, Gamma-proteobacteria and Flavobacteria are often reported as the dominant free-living bacterioplankton during algal bloom (reviewed in Buchan et al., 2014). It is still unclear, however, if phytoplankton-associated bacteria undergo the same pattern and if bacterial composition and succession is being influenced by the algal species in bloom or if the phytotoxins produced by harmful algae also play a role in structuring bacterial communities during algal bloom.

Many bacteria associate with phytoplankton (Schafer et al., 2002; Grossart et al., 2005; Sison-Mangus et al., 2014) and are often seen attached on healthy or dying cells (Kaczmarska et al., 2005; Gardes et al., 2011; Mayali et al., 2011; Smriga et al., 2016), as they feed off on the released dissolved organic matter, breaking down the dead algal cell (Bidle and Azam, 1999) or actively killing the phytoplankton (Mayali and Azam, 2004). Phytoplankton cultivates close associations with bacteria as imposed by their dependence on vitamins, recycled nutrients, photooxidation of assimilable iron or amino acids (Croft et al., 2005; Amin et al., 2009, 2015). On the other hand, phytoplankton varies in terms of biochemical composition, organic excretions (Reitan et al., 1994; Hellebust, 1980; Bjornsen, 1988) or phytotoxins (Doucette et al., 1998), which may act as selective agents for bacterial types with algal-attached lifestyle. It is therefore interesting to determine if bloom-forming phytoplankton species can also facilitate the dominance of different bacterial groups during bloom events.

In this study, we focus on the domoic acid producing *Pseudo-nitzschia* (Pn) and studied the phytoplankton-associated bacterial community from four independent natural bloom events. We asked what bacterial groups dominate in bloom events formed by different *Pseudo-nitzschia* species. We determined how bacterial composition changes before, during and after the decline of *Pseudo-nitzschia* bloom, as it was replaced or co-dominated by other phytoplankton species. Aside from phytoplankton biomass (as chlorophyll *a*), we also looked at environmental variables such as nutrients (nitrate, phosphate, silicate, urea, and ammonium), and temperature to determine if these physical factors also play a role in bacterial succession. Our results indicated that bacterial composition and structure are strongly influenced by the *Pseudo-nitzschia* species in bloom and domoic acid can play a role in limiting bacterial diversity.

## Materials and Methods

### Plankton Tow, Bacteria Sampling and Environmental Data Collection and Processing

#### Sampling Site

The samples used in the study were subsamples from the weekly monitoring of harmful algal bloom adjacent to Santa Cruz Municipal Wharf (36.9633°N, 122.0172°W) in Monterey Bay, California, one of the ocean observing sites in Central and Northern California (CeNCOOS). The site was established to monitor the presence, abundance and population dynamics of red tide forming species, where weekly monitoring of nutrients, environmental parameters, phytoplankton abundance, chlorophyll *a* and fecal indicator bacteria are measured. Water condition in Monterey Bay is characterized by seasonal upwelling that brings low temperature, high salinity nutrient-rich water from approximately February to August, followed by an oceanic period characterized by upwelling relaxation in August to mid-November. In winter (mid November–mid February), the Davidson Current surfaces along the coast and brings in relatively warm, high salinity, nutrient-rich water (Skogsberg, 1936; Skogsberg and Phelps, 1946; Wyllie and Lynn, 1971; Pennington and Chavez, 2000). Upwelled water together with agricultural run-offs from numerous watersheds feed nutrients into the bay to support massive phytoplankton blooms (Kudela and Chavez, 2004; Kudela et al., 2008; Fischer et al., 2014). Different *Pseudo-nitzschia* species bloom in regular pattern in the bay since their initial discovery in 1991 (Buck et al., 1992; Scholin et al., 2000; Lane et al., 2009).

#### Water and Phytoplankton Sampling

Vertical phytoplankton tows are normally collected in the early morning at an integrated depth of 1–10 ft depth (5 × 10 ft vertical effort) using a 20 μm mesh with a cod end volume of 300 mL. A 2-L Niskin bottle was used to collect water samples at the same integrated depth and used for nutrients and chlorophyll analysis. Temperature was measured immediately after water collection. For this study, opportunistic sampling was carried out at different phases of the bloom, from the beginning and end of the bloom in November to December 2010 (Fall) and peak and demise in March (Spring), July and August 2011 (Summer). Bloom phases were categorized based on total number of *Pseudo-nitzschia* cells per liter and are as follows: No bloom – 0 to 10000; Low Bloom – 10, 001 to 30,000; Medium bloom – 30,001 – 100, 000; High Bloom – 100001 to 300000; Highest Bloom- >300, 000. Closure of shellfish harvesting commonly occurs when DA-producing *Pseudo-nitzschia* bloom is seen at 100, 000 cells L^1^ (Bates et al., 1998).

#### Algal Counting, Domoic Acid, Chlorophyll and Nutrients Measurement

Algal counts were initially assessed microscopically based on relative percentage abundance followed by counting of total *Pseudo-nitzschia* cells by microscopy and counting of toxic *Pseudo-nitzschia* species using DNA fluorescent in-situ hybridization (FISH) (Miller and Scholin, 1998). Domoic acid in bloom samples were measured using LC/MS method as described in Lane et al. (2010). Briefly, water samples were filtered on GFF filters and stored in -80°C. Ice-thawed filters were submerged in 3 mL 10% MeOH and intermittently sonicated on ice for 30 sec at an output power of <8 Watts. The thick suspension was syringe filtered with 0.22 μm Millex PVDF membrane (EMD Millepore, USA), and stored in -80°C until further processing. DA samples were purified prior to LC/MS analysis. Steps are as follows: DA samples were first prepared by adding 0.5 mL of formic acid and MeOH diluted with Milli-Q water (2:5:93 ratio) and briefly vortexed. The solid phase extraction (SPE) columns (Agilent, USA) were first conditioned with 10-mL 100% MeOH followed by 10-mL Milli-Q water under gentle vacuum of 10Pka, and not letting the SPE columns to dry. After addition of DA samples to the conditioned columns, the sample tubes were rinsed with 4-mL 0.5% aqueous formic acid, and combined with the samples already in the columns. An additional step of adding 4-mL 0.15% formic acid was carried out. DA in the columns were recovered with 3 mL 50% MeOH and transferred to a pre-weighed glass vial for storage at 4°C until analysis of DA via LC/MS (described in Lane et al., 2010). Nutrient samples (nitrate + nitrite hereafter referred to here as nitrate, phosphate and silicate) pre-filtered in GF/F filters (nominal size of 0.7 μm) were analyzed using a Lachat QuikChem 8500 Flow Injection Analyst System and Omnion 3.0 software (Lachat Instruments; Hach Company, Loveland, CO, USA) following the method described in Smith and Bogren (2001) and Knepel and Bogren (2002). Ammonium and urea were analyzed with the method of Holmes et al. (1999) and Price and Harrison (1987), respectively. Chlorophyll *a* analysis was carried out following the non-acidification method described in Welschmeyer (1994).

#### Bacteria Sampling

Phytoplankton tow samples were first pre-filtered with 300-μm mesh net (bleached and UV-exposed) to remove debris and zooplankton. Three of 30-mL tow samples were filtered with sterile 5 μm Durapore filters (EMD Millipore, USA) and washed with sterile seawater to filter out unassociated bacteria. Algae were collected by resuspending filters in 10 ml of PCR water. Samples were then pelleted via centrifugation at 3000 × *g* for 10 min. Algal pellets were finally resuspended in 500 μL of sterile TE buffer and stored at -80°C. For sequencing, raw samples were sent to Research and Testing Laboratory (Lubbock, TX, USA) for 454 pyrosequencing using the universal primers Yellow939F and Yellow1492R. Sample preparation for sequencing and sequencing methods are described in Sison-Mangus et al. (2014).

### *Pseudo-nitzschia* Isolation and Genotyping

Single cells of *Pseudo-nitzschia* were manually isolated from bloom samples with a microcapillary tip from a sterile glass Pasteur pipette. Prior to cell isolation, an aliquot of the pre-filtered tow sample was filtered with 100 μm nylon mesh (bleached and UV-sterilized) and washed two times with autoclaved-sterilized 0.2 μm filtered seawater (FSW) to remove unassociated bacteria. Algal suspension was diluted with FSW. Isolated cells were washed 10 times with FSW, inoculated in F/10-Se medium and incubated under growth conditions of 12:12 photoperiod, 15°C and 80 μE m^-2^ in an algal incubator. Successful isolates were transferred in F/2-SE medium for maintenance. The *Pseudo-nitzschia* cultures were identified by morphology using light microscopy and genotyped by sequencing the 18S rRNA gene.

*Pseudo-nitzschia* genomic DNA was extracted with PowerSoil DNA Isolation Kit (MoBio Laboratories Inc., Solana Beach, CA, USA). *P. pungens* and *P. fraudelenta* were amplified with primer pairs 1360F (5′-GCGTTGAT/ATACGTCCCTGCC- 3′) and ITS055R (5′-CTCCTTGGTCCGTGTTTCAAGACGGG-3′), while *P. australis* and *P. multiseries* was amplified with primer pairs 18S-F (5′-CTGCGGAAGGATCATTACCACA-3′) and ITS055R using EconoTaq DNA Polymerase (Lucigen, Middleton, WI, USA) under the following PCR conditions: 94°C for 2 min, 30 cycles of 94°C for 1 min, 55°C for 1 min, and 72°C for 1.5 min and 72°C for 10 min extension. The products were sent to a service facility (Laragen Inc, Culver, CA, USA) for direct sequencing after labeling with the Big Dye Terminator with AmpliTaq FS Sequencing Kit (Applied Biosystems, Foster City, CA, USA) using the PCR primers as sequencing primers.

### 454 Sequence Data Processing

Short sequence reads (length <150 bp), low quality sequences (score <25; Huse et al., 2007) and any non-bacterial ribosome sequences and chimeras (Gontcharova et al., 2010) were removed prior to sequence analysis using Quantitative Insights Into Microbial Ecology (QIIME 1.8) pipeline (Caporaso et al., 2010b). Uclust (Edgar, 2010) was used in picking operational taxonomic units (OTUs) based on clustering sequences at 97% similarity. Representative sequences were aligned using Pynast (Caporaso et al., 2010a) using Greengenes core alignment as a reference (DeSantis et al., 2006). OTUs were assigned based on RDP classifier 2.2 (Wang et al., 2007), while the assignment of abundant bacterial taxon at the genus level was performed using BLAST [Altschul et al., 1990, *E*-value 10_20 (megablast only); minimum coverage 97%; minimum pairwise identity 90–97%]. Singletons and chloroplast sequences were filtered from the OTU table prior to bacterial diversity analyses. A phylogenetic tree of the OTUs was generated and viewed with Fast Tree 2.1.3 (Price et al., 2010).

The raw sequencing reads have been deposited at the NCBI Short Read Archive under BioProject ID PRJNA329303 with the following BioSample accession numbers: SAMN05410841 (Tow_11.17.10), SAMN05410842 (Tow_12.01.10), SAMN0541084 (Tow_12.08.10), SAMN05410844 (Tow_12.15.10), SAMN05410845 (Tow_12.22.10), SAMN05410846 (Tow_03.16.11), SAMN05410847 (Tow_03.23.11), SAMN05410848 (Tow_07.06.11), SAMN05410849 (Tow_07.13.11), SAMN05410850 (Tow_08.24.11), SAMN05410851 (Tow_08.31.11).

### Statistical Analysis

Correlation tests using multivariate non-parametric Spearman rank’s correlation coefficient and Student’s *t*-test were carried out using the software JMP 12.0.

## Results

### Phytoplankton Community at Different Phases of *Pseudo-nitzschia* Bloom

**FIGURE 1:**
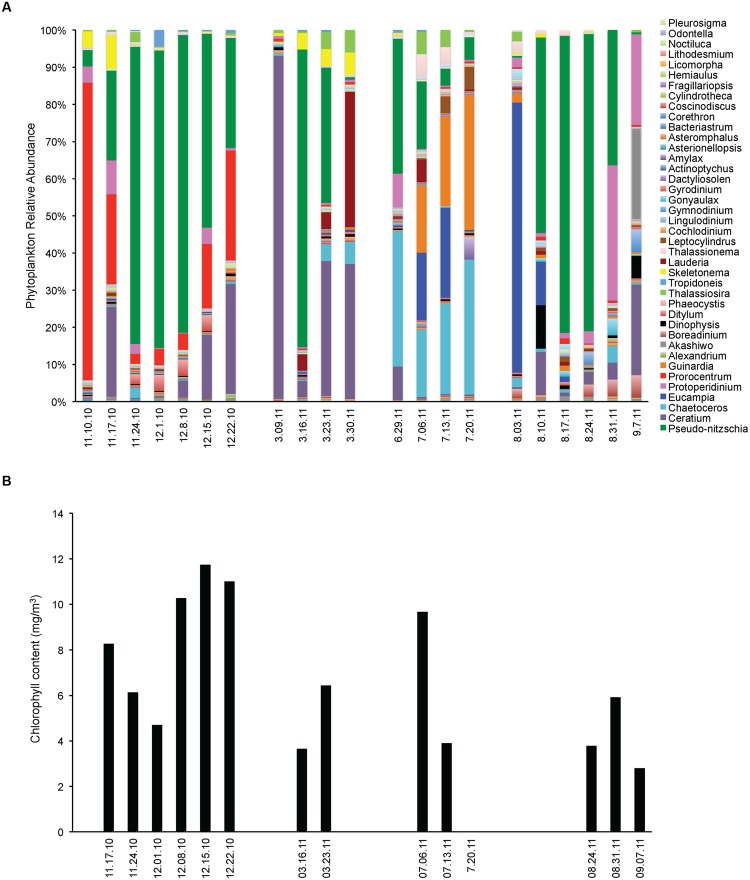
**Phytoplankton community assemblage before, during and after *Pseudo-nitzschia* bloom events in 2010 and 2011. (A)** Phytoplankton relative abundance for each algal group assessed by microscopy. **(B)** Phytoplankton biomass expressed as total chlorophyll content.

**FIGURE 2:**
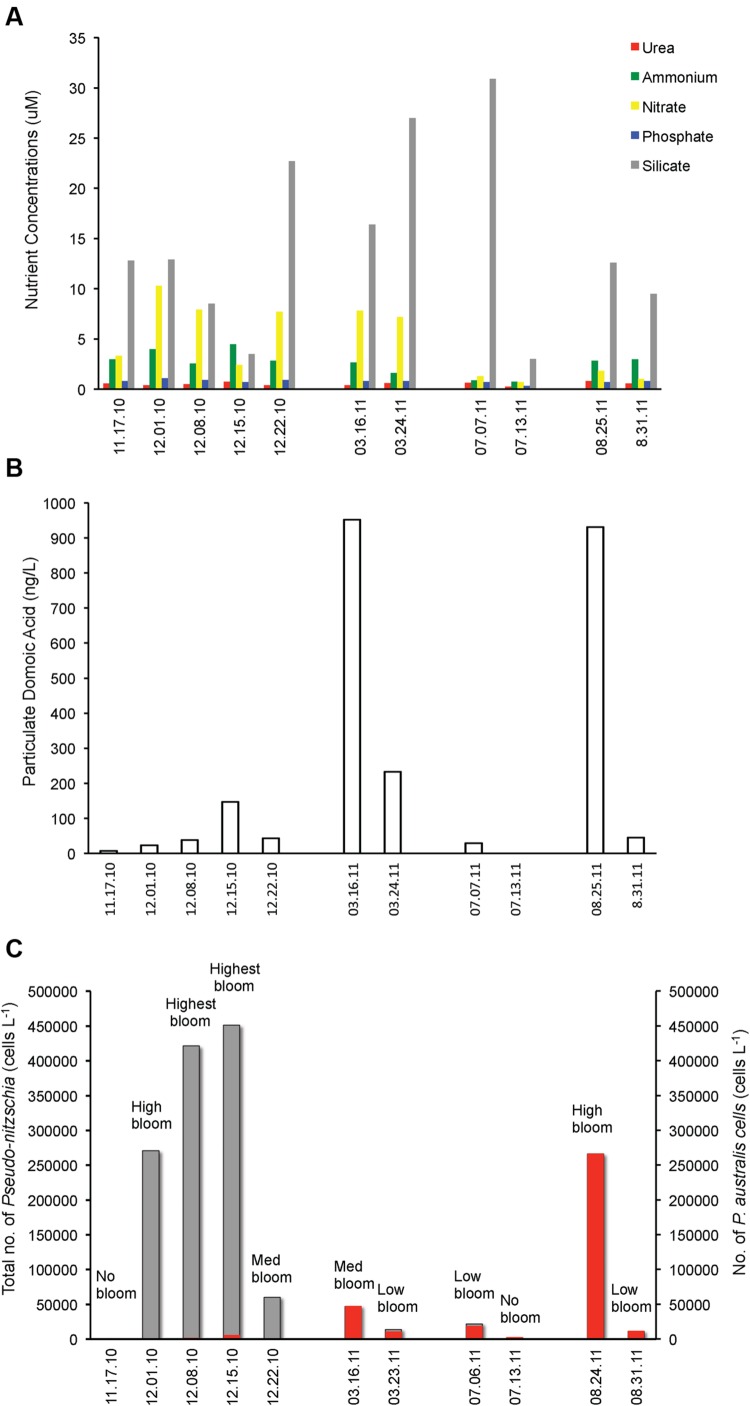
***Pseudo-nitzschia* cell numbers, domoic acid (DA) and nutrient concentrations before, during and after *Pseudo-nitzschia* bloom events in 2010 and 2011. (A)** Urea, ammonium, nitrate, phosphate, and silicate concentrations. **(B)** Particulate domoic acid content. **(C)** Total number of *Pseudo-nitzschia* cells (gray) superimposed with the *P. australis* cell numbers (red).

Opportunistic sampling was done in Santa Cruz Municipal Wharf during the occurrence of *Pseudo-nitzschia* bloom. In mid-November 2010, phytoplankton diversity was high with the community being co-dominated by *Pseudo-nitzschia*, *Prorocentrum* and *Ceratium* (**Figure 1**). The assemblage changed into mostly *Pseudo-nitzschia* as the massive bloom progressed for 4 weeks. At the end of the bloom at week 5, there was an equal presence between *Pseudo-nitzschia* and dinoflagellates. Single algal cell isolations followed by genotyping indicated that *P. fraudulenta* dominated the *Pseudo-nitzschia* community and FISH probing and counting indicated that *P. australis* were also present in small number (**Figure 2C**).

In 2011, an early spring bloom of *P. australis* occurred in mid-March, but this bloom did not last as the population of the dinoflagellate *Ceratium* co-dominated the following weeks. In July 2011, a small bloom of *P. australis* was seen together with *Chaetoceros* and *Eucampia*, but noticeably decreased the following weeks and was replaced by higher diversity of phytoplankton with the co-dominance of *Guinardia*, *Chaetoceros* and *Eucampia*. In August 2011, a massive *P. australis* bloom occurred that lasted for 3 weeks. The algal community shifted and co-dominated both by the dinoflagellates *Protoperidinium* and *P. australis* during the fourth week, but phytoplankton biomass has decreased. *P. australis* were the dominant *Pseudo-nitzschia* species in 2011 (**Figure 2C**), with a small number of *P. multiseries* (3450 cells/ L) seen on March 23, 2011. Chlorophyll levels for the data gathered during opportunistic sampling showed that overall phytoplankton biomass range from 2.8 to 11.7 mg m^-3^, with the highest chlorophyll level observed in 2010 during the bloom dominated by *P. fraudulenta*, where positive correlation existed (Spearman rho = 0.76, *p* < 0.006). Temperature range during late Fall 2010 sampling was between 11–14°C, while it was 12°C during Spring 2011 and 14–15°C in the Summer 2011. There was no correlation seen between temperature and chlorophyll level, or between temperature and total *Pseudo-nitzschia* cells, *Pseudo-nitzschia* species or DA. Additionally, a negative correlation of temperature with nitrate was seen (Spearman rho = -0.84, *p* < 0.001).

### Nutrients, *Pseudo-nitzschia* Number and DA Level Are De-coupled during *Pseudo-nitzschia* Algal Bloom

We looked at the correlation between nutrients and total *Pseudo-nitzschia* cell number and the presence of toxic *Pseudo-nitzschia* species (**Figure 2**). Interestingly, none of the nutrients (urea, ammonium, nitrate, phosphate, and silicate) were correlated with cell abundance of *Pseudo-nitzschia* in a natural bloom and the five nutrients did not show any correlation with *P. fraudulenta*, *P. australis*, *P. multiseries* cell numbers (data not shown). Notably, DA level was positively correlated only with *P. australis* cell numbers (Spearman rho = 0.69, *p* < 0.02) but not with total *Pseudo-nitzschia* number or with *P. fraudulenta* cell number, even though this low-toxin producing species was present in very high numbers in 2010 bloom (**Figures 2B,C**). Likewise, temperature did not show any correlation with total *Pseudo-nitzschia* cell numbers, *P. fraudulenta* or *P. australis* cell numbers or with any of the aforementioned parameters, except for nitrate, to which it showed negative correlation (Spearman rho = -0.84, *p* < 0.001).

### High DA-Producing *P. australis* Negatively Affects Bacterial Diversity in Natural Bloom

A total of 83,284 clean 16S rDNA sequences was obtained from 11 samples belonging to four *Pseudo-nitzschia* bloom events captured at different bloom phases (before, during and at the decline phases in 2010 and during and at decline phases in 2011). These sequences were assigned into 4732 OTUs (>97% ID), but reduced to 2621 OTUs after removing singletons and chloroplast sequences. The remaining OTUs were analyzed for bacterial diversity in every sample. Statistical analysis indicated that there was no significant correlation between phytoplankton biomass (i.e., chlorophyll *a*) and bacterial diversity (measured as Observed number of OTUs, Phylogenetic diversity, Shannon and Chao indices) suggesting that total phytoplankton biomass did not influence bacterial diversity (**Table 1**).

**Table 1:** Correlations between environmental variables and bacterial diversity as measured by four diversity indices.

	Spearman rho	*p*-level
**Phyto biomass (chl *a*) vs. bacterial diversity**		
No. of OTUs	0.40	0.223
Shannon index	0.08	0.811
Phylo. diversity index	0.40	0.223
Chao index	0.40	0.223
**Total no. of Pn cells vs. bacterial diversity**		
No. of OTUs	0.14	0.689
Shannon index	-0.13	0.709
Phylo. diversity index	0.15	0.670
Chao index	0.14	0.689
***P. australis* no. vs. bacterial diversity**		
No. of OTUs	-0.85	0.001
Shannon index	-0.62	0.043
Phylo. diversity index	-0.85	0.001
Chao index	-0.85	0.001
***P. fraudulenta* no. vs. bacterial diversity**		
No. of OTUs	-0.90	0.037
Shannon Index	-0.70	0.1881
Phylo. diversity index	-1.0	<0.0001
Chao index	-0.90	0.037
**Temperature vs. bacterial diversity**		
No. of OTUs	-0.65	0.032
Shannon index	-0.78	0.005
Phylo. diversity index	-0.55	0.077
Chao index	-0.65	0.032
**Nitrate vs. bacterial diversity**		
No. of OTUs	0.70	0.017
Shannon index	0.72	0.013
Phylo. diversity index	0.64	0.035
Chao index	0.70	0.017
**Phosphate vs. bacterial diversity**		
No. of OTUs	0.65	0.032
Shannon index	0.60	0.052
Phylo. diversity index	0.55	0.082
Chao index	0.65	0.032

**FIGURE 3:**
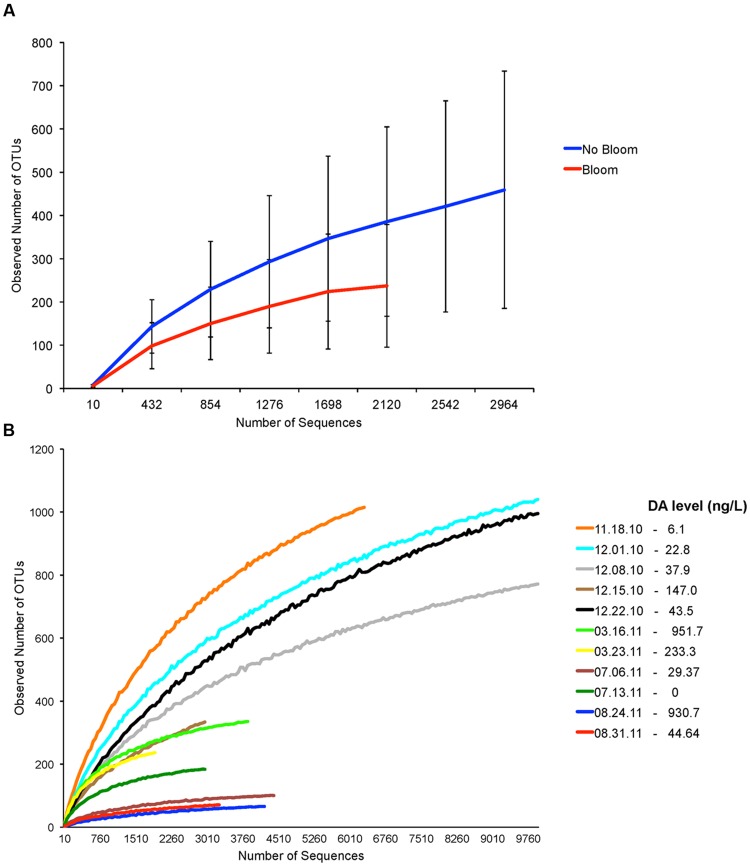
**Assessment of bacterial diversity based on number of Operational Taxonomic Units (OTUs) from samples before, during and after *Pseudo-nitzschia* bloom events in 2010 and 2011. (A)** Rarefaction curves generated from samples with no *Pseudo-nitzschia* bloom and *Pseudo-nitzschia* bloom. **(B)** Individual rarefaction curves generated for each sample. Particulate domoic acid level is shown for each sample in the legend. Low-toxin producer *P. fraudulenta* dominated in 2010 bloom while the high-toxin producer *P. australis* dominated in 2011 blooms.

We determined if bacterial diversity was specifically affected by the presence of *Pseudo-nitzschia* bloom. Although bacterial diversity was lower during *Pseudo-nitzschia* bloom (**Figure 3A**), comparison of four bacterial indices (calculated at 2000 sequences depth) between *Pseudo-nitzschia* bloom and no-bloom suggested that bacterial diversity was not significantly different between the two events (Student’s *t*-test, *p* > 0.05). However, the correlation analysis at the *Pseudo-nitzschia* species level indicated that the presence of high DA-producing *P. australis* negatively affected bacterial diversity (**Table 1**). Bacterial diversity was generally lower when the *Pseudo-nitzschia* bloom was dominated by *P. australis* versus *P. fraudulenta* (**Figure 3B**). Because low DA-producing *P. fraudulenta* were only present in 2010, correlation analysis for *P. fraudulenta* number versus bacterial diversity was carried out on this small data set. We found that as *P. fraudulenta* increased in number, bacterial diversity also declined (**Table 1**). Also, closer inspection indicated that bacterial diversity was lower as DA accumulated, due to increasing number of *P. fraudulenta* and the combined presence of *P. australis* (**Figures 3B** and **2C**). Bacterial diversity also negatively correlated with temperature while it positively correlated with phosphate and nitrate concentrations (**Table 1**).

### Dominant Bacterial Groups Vary with Different *Pseudo-nitzschia* Bloom Events

**FIGURE 4:**
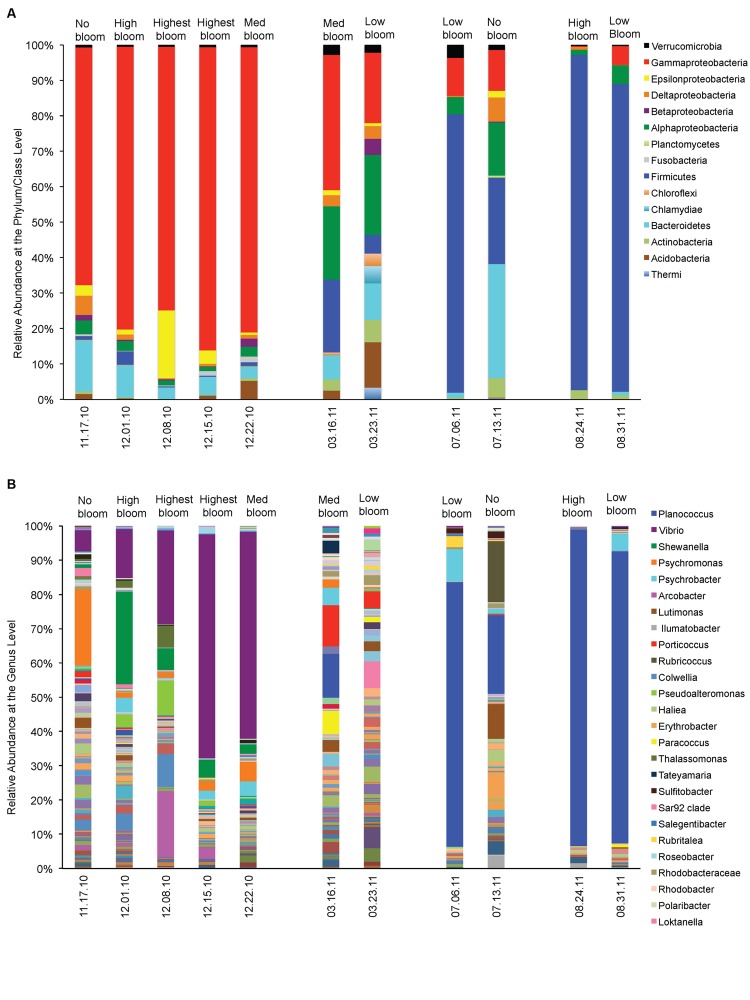
**Relative abundances of phytoplankton-associated bacteria before, during and after *Pseudo-nitzschia* bloom events in 2010 and 2011. (A)** Relative abundance of bacteria at the phylum/ class level. **(B)** Relative abundance of bacteria at the Genus level.

We asked which bacterial groups dominate before, during or at the decline phase of *Pseudo-nitzschia* bloom and looked at other environmental variables that may facilitate their dominance. At least ten bacterial phyla (or class) were frequently found associating with phytoplankton during non-bloom events (**Figure 4**). However, during *Pseudo-nitzschia* bloom events, only a few bacterial phyla dominated the bacterial assemblages (**Figure 4A**) and these bacterial assemblages changed depending on the dominant *Pseudo-nitzschia* species that comprised the bloom. In 2010, Gamma-proteobacteria (65–85%) were prevalent when a low-toxic species, *P. fraudulenta*, dominated the *Pseudo-nitzschia* populations (Spearman rho = -0.79, *p* < 0.004) and decreased in relative abundance during *P. australis* bloom (0–38%). These bacterial groups were also correlated with nitrate concentrations (Spearman rho = -0.64, *p* < 0.035). The Firmicutes phyla (20–95%) dominated in 2011 during high toxin blooms and were highly correlated with *P. australis* cell number (Spearman rho = -0.72, *p* < 0.013). Bacteroidetes (0–32%) and Epsilonbacteria (0–19%) groups were mostly present in the background but were not significantly correlated with the environmental conditions studied; Bacteroidetes, however, had higher relative abundance during non-Pn bloom events (14–32%) than during Pn bloom (0–9%; **Figure 4A**). Similarly, Alpha-proteobacteria were also always present but their abundance was negatively correlated with the total number of *Pseudo-nitzschia* cells especially in the presence of high number of *P. fraudulenta* (1–3% vs. 15–20% during low or no Pn-bloom; Spearman rho = -0.77, *p* < 0.006). On the contrary, Fusobacteria (1–2%) were only present in 2010 bloom and positively correlated with *P. fraudulenta* abundance (Spearman rho = -0.79, *p* < 0.004). Bacterial groups that were present but in low relative abundance such as the Chloroflexi (0–3%), Acidobacteria (0–12%), and Betaproteobacteria (0–4%) groups were negatively correlated with temperature (Spearman rho = -0.60–0.79, *p* < 0.05). Actinobacteria (0–6%), another low abundant bacterial group, was only present when there was low number of *Pseudo-nitzschia* cells in the water (in 2011) (Spearman rho = 0.67, *p* < 0.02). The presence of the very low abundant group Verrucomicrobia (1–4%) and Planctomycetes (0–1%) were not correlated with any of the aforementioned environmental parameters.

At the genus level, *Vibrio* spp. was the Gamma-proteobacteria that dominated the *Pseudo-nitzschia* bloom in 2010 (**Figure 4B**). *Vibrio* increased in relative abundance (6–65%) as the bloom of *Pseudo-nitzschia* progressed into its peak, and positively correlated with the increase of *P. fraudulenta* cells, the abundant *Pseudo-nitzschia* species in 2010 bloom (Spearman rho = 0.8913, *p* < 0.0002). *Vibrio* was also positively influenced by phytoplankton biomass (chl) (Spearman rho = 0.78, *p* < 0.004) but it did not dominate in *P. australis* bloom in 2011. *Planococcus* spp. (12–93%), bacteria from the Firmicutes group, was positively correlated with the bloom of *P. australis* in 2011 (Spearman rho = 0.60, *p* < 0.05), where it dominated in three independent *P. australis* blooms. Temperature might have also significantly influenced its dominance (Spearman rho = 0.7426, *p* < 0.009).

*Shewanella* (1–27%), the third most abundant bacteria in the samples, is an Alpha-proteobacteria that was present in 2010 bloom and showed positive correlations with *P. fraudulenta* (Spearman rho = 0.90, *p* < 0.0002), together with nitrate and phosphate (Spearman rho = 0.84 and 0.71, *p* < 0.01). *Erythrobacter* (0–8%), another Alpha-proteobacteria, was present only in 2011 blooms and was negatively correlated with nitrate and ammonium (Spearman rho = -0.78 and -0.68, *p* < 0.03). The abundant Gamma-proteobacteria bacteria, *Psychromonas* (1–21%), was mostly seen in 2010 and its presence was not significantly influenced by any of the environmental variable studied other than *P. fraudulenta* cell number (Spearman rho = 0.65, *p* < 0.03). *Psychrobacter* (0–9%), another Gamma-proteobacteria, was ubiquitous in many samples but was not specifically influenced by any of the environmental variables. The *Lutimonas* bacteria and other Flavobacteriaceae (0–10%) also did not show any correlations with any of the environmental variables. *Rubricoccus* (0–18%), another Bacteroidetes, showed positive correlation with temperature (Spearman rho = 0.62, *p* < 0.04). The Epsilonbacteria, *Arcobacter* (0.4–19%), was abundant in 2010 blooms and was positively correlated with *P. fraudulenta* and nitrate (Spearman rho = 0.85 and 0.67, *p* < 0.02, respectively).

## Discussion

This study followed phytoplankton-associated bacterial community composition and succession from four natural *Pseudo-nitzschia* blooms in the same biogeographical location sampled before, during and after bloom and looked at how harmful algal bloom species and phytotoxin, domoic acid, influence phytoplankton-associated bacterial communities. Our study showed that the composition of the bacterial communities is driven by the phytoplankton species that comprised the bloom, while some less-abundant bacterial genera are also driven by phytoplankton biomass, temperature and nutrients (specifically nitrate and phosphate). Two species of *Pseudo-nitzschia* were found to dominate in the four bloom events, with the low-DA producing *P. fraudulenta* dominating in late Fall 2010 bloom (with a mix of small numbers of *P. australis*), and the high-DA producing *P. australis* dominating in three blooms in the spring, mid-summer and late summer of 2011. The dominant bacterial group changed with the dominant algal species that comprised the algal bloom. Moreover, with each bloom phase shift (increasing or decreasing of *Pseudo-nitzschia* cell numbers), these dominant bacterial groups also followed the same trend with the algal shift, that is high *P. fraudulenta* or *P. australis* cell number also increased the abundance of the respective dominant bacterial group. In this study, our approach did not allow us to study the composition of the bacterial community associated to a specific algal species but rather from a community of phytoplankton species that was dominated by *Pseudo-nitzschia* during bloom events. The Gamma-proteobacteria *Vibrio* spp., for instance, increased in relative abundance as *P. fraudulenta* cells increased in number, while the Firmicutes bacteria, *Planoccocus* spp. decreased in abundance as *P. australis* decreased in abundance. *Vibrio* spp. are known to associate with phytoplankton and zooplankton (Tamplin et al., 1990; Hsieh et al., 2007; Main et al., 2015) while *Planococcus* spp. are often isolated from *Pseudo-nitzschia* and are reported to be members of *Pseudo-nitzschia* microbiome (Sison-Mangus et al., 2014).

We also observed that when *P. australis* were replaced or co-dominated by other diatoms (*Chaetoceros*, *Eucampia*) or dinoflagellates (*Guinardia*, *Protoperidinium*), the bacteria *Planococcus* spp. was also replaced or co-dominated by the abundance of other bacterial groups from Bacteroidetes, Gamma- and Alpha-proteobacteria. This suggests that specific bacteria and phytoplankton groups can influence each other and their abundance does not only depend on phytoplankton biomass abundance during bloom but that the relationship can also be dictated by the diversity of algal species in the phytoplankton community. Rooney-Varga et al. (2005) have reported similar findings where the phytoplankton community is responsible for the significant amount of variability in the attached-bacterial community composition. Just like in their study, we did not see any correlation between bacterial diversity and phytoplankton biomass (i.e., chlorophyll *a*, **Table 1**), hence a closer look at the phytoplankton species composition during and after phytoplankton blooms is necessary if we want to understand the factors that facilitate the dominance of phytoplankton-associated bacterial groups and their role in the degradation of phytoplankton-derived organic matter.

Temperature, nitrate and phosphate seem to play a role in facilitating the abundance of some bacterial groups, but our data set is not enough to resolve if these abiotic factors can drive bacterial succession. Indeed, these physical factors affect the metabolism of phytoplankton and influences algal growth, which ultimately can influence phytoplankton-associated bacterial composition and diversity. In our study, we found no correlation between phytoplankton biomass, *Pseudo-nitzschia* cell number, nutrients (urea, ammonium, phosphate, silicate) and temperature, but nitrate and temperature did show negative correlation. Hence, a longer time series is suggested to give sufficient evidence to confirm the direct influence of these physical factors on bacterial community composition and succession.

Our current study showed that the neurotoxin domoic acid tend to limit bacterial diversity or bacterial OTU richness. For instance, the abundance of high-DA producing *P. australis* depressed bacterial diversity when it dominated the bloom in 2011. *P. fraudulenta*, a low-DA toxin producing *Pseudo-nitzschia*, also depressed bacterial diversity as DA increased when *P. fraudulenta* increased in cell number, but the bacteria diversity is not as low as that observed in *P. australis* bloom (**Figure 3B**). Interestingly, the presence of *P. australis* (albeit in small number), with *P. fraudulenta* in the December 15, 2010 bloom sample increased the DA level and drove the bacterial diversity further down (**Figure 3B**). These field-based results agreed with our past laboratory study, where we found a higher bacterial diversity harbored by cultured *P. fraudulenta* strains and a lower bacterial diversity in cultured *P. australis* strains (Sison-Mangus et al., 2014). This lends strong support to the idea that DA can structure *Pseudo-nitzschia*-associated bacterial communities. Domoic acid may act as a selective agent that can structure bacterial communities either by serving as a nutrient source for associated bacteria that can effectively assimilate DA or the toxin may act as deterrent to other associative bacteria. Previously, domoic acid has been hypothesized to be anti-bacterial (Bates et al., 1995) but concrete evidence is currently lacking to support this hypothesis.

Current efforts are now focused on understanding which bacterial groups can degrade various types of phytoplankton-derived organic matter. The pervading principle is that bacterial diversity and composition is largely driven by the availability of organic material from phytoplankton biomass, and the bacteria that can degrade this copious amount of algal-derived organic material will get selected and proliferate in the marine environment. Landa et al. (2016), for instance, reported that bacterial groups traditionally known for being algal bloom-associated, have dominated the pool of enriched bacterial groups observed from artificially iron-induced phytoplankton blooms in the Southern Ocean. Recent studies have also looked at the genome of free-living bacteria that are dominant during phytoplankton bloom, which comprises mostly of Flavobacteriaceae, *Roseobacters* and Gamma-proteobacteria to determine their genetic capability of degrading phytoplankton-derived material (Teeling et al., 2012, 2016; Klindworth et al., 2014). We can use the same argument on the selection process undergone by bacterial groups that develop an associative lifestyle with phytoplankton. Associated bacteria are proximally at an advantage to readily access phytoplankton exudates when the algal host secretes them and can access the algal organic material for consumption once the host cell is dead, a niche filled by copiotrophic bacteria that may have led to the observation that attached bacteria are distinct and showed little to high diversity than free-living bacteria (Delong et al., 1993; Acinas et al., 1999; Mohit et al., 2014). Association of bacteria with phytoplankton can evolve into mutualistic partnership such as that observed between *Sulfitobacter* and *P. multiseries* (Amin et al., 2015) or to commensal-parasitic relationship between *Phaeobacter* and *Emiliania huxleyi* (Seyedsayamdost et al., 2011), or host-specificity between *Pseudo-nitzschia* and its microbiome (Sison-Mangus et al., 2014). To understand the evolution of attached lifestyle among phytoplankton-associated bacteria, comparative genome analysis complemented with physiological and biochemical approaches are inviting if we want to gain a mechanistic insight of their evolutionary strategy. Similarly, field-based studies complemented with laboratory manipulations are required to understand the various influences of associated bacteria on phytoplankton physiology such as those related to toxin production, organic material production and recycling and algal bloom formation.

## Author Contributions

MSM and SJ conceived the project. MSM and RK executed the project, MSM and SM did the data analysis. MSM, SJ, and RK wrote the paper.

## Conflict of Interest Statement

The authors declare that the research was conducted in the absence of any commercial or financial relationships that could be construed as a potential conflict of interest.
